# DETIRE: a hybrid deep learning model for identifying viral sequences from metagenomes

**DOI:** 10.3389/fmicb.2023.1169791

**Published:** 2023-06-16

**Authors:** Yan Miao, Jilong Bian, Guanghui Dong, Tianhong Dai

**Affiliations:** College of Computer and Control Engineering, Northeast Forestry University, Harbin, China

**Keywords:** metagenome, viral identification, deep learning, graph convolutional network, codon embedding

## Abstract

A metagenome contains all DNA sequences from an environmental sample, including viruses, bacteria, archaea, and eukaryotes. Since viruses are of huge abundance and have caused vast mortality and morbidity to human society in history as a type of major pathogens, detecting viruses from metagenomes plays a crucial role in analyzing the viral component of samples and is the very first step for clinical diagnosis. However, detecting viral fragments directly from the metagenomes is still a tough issue because of the existence of a huge number of short sequences. In this study a hybrid Deep lEarning model for idenTifying vIral sequences fRom mEtagenomes (DETIRE) is proposed to solve the problem. First, the graph-based nucleotide sequence embedding strategy is utilized to enrich the expression of DNA sequences by training an embedding matrix. Then, the spatial and sequential features are extracted by trained CNN and BiLSTM networks, respectively, to enrich the features of short sequences. Finally, the two sets of features are weighted combined for the final decision. Trained by 220,000 sequences of 500 bp subsampled from the Virus and Host RefSeq genomes, DETIRE identifies more short viral sequences (<1,000 bp) than the three latest methods, such as DeepVirFinder, PPR-Meta, and CHEER. DETIRE is freely available at Github (https://github.com/crazyinter/DETIRE).

## 1. Introduction

High-throughput sequencing or next-generation sequencing (NGS) technology, which makes it possible to obtain all nucleotide sequences directly from environmental samples, has played important roles in many fields, such as pathogen detection (Wu et al., 2015; Miao et al., 2022) and human disease analysis (Georg et al., 2014; Santiago-Rodriguez and Hollister, 2019; Zhu et al., 2019). In these applications, detecting viruses from metagenomic sequences becomes more and more essential because it is the first step in the analysis of viruses (Bonhoeffer and Sniegowski, 2002). However, it is still a quite difficult task because of their relatively low biomass compared with those of bacteria and high mutation rates.

To overcome this challenge, several methods have been proposed to identify viruses from metagenomes in the past few years and can be categorized into similarity-based, machine learning-based, and deep learning-based methods. Similarity-based methods generally map a query sequence to a reference dataset and recognize it as the one with the highest similarity score (Roux et al., 2011, 2015; Wommack et al., 2012; Wood and Salzberg, 2014; Buchfink et al., 2015; Kim et al., 2016; Rampelli et al., 2016; Truong et al., 2016; Paez-Espino et al., 2017; Vilsker et al., 2019). These methods, however, suffer from long execution time during the mapping process, and hardly detect short viral sequences because of the limited features they have. Different from the similarity-based methods, machine learning-based methods could extract human-designed features from DNA sequences and classify them by a well-trained classifier, such as VirFinder (Ren et al., 2017), MARVEL (Amgarten et al., 2018), and VirSorter2 (Guo et al., 2021). Although these methods have the ability to produce more accurate results, the features have to be designed artificially, and the performance in identifying short viral sequences is relatively poor.

With the great success of deep learning methods in the past few years, several deep learning-based methods have been proposed to identify viruses from metagenomes. Long-short term memory (LSTM) network and convolutional neural network (CNN) are the most commonly used models. For example, ViraMiner (Tampuu et al., 2019), VirNet (Abdelkareem et al., 2018), and RNN-VirSeeker (Liu et al., 2022) utilize a single LSTM network to learn the interconnections between each part in a one-hot encoded sequence; DeepVirFinder (Jie et al., 2020), PPR-Meta (Fang et al., 2019), and CHEER (Shang and Sun, 2021) establish a single CNN to extract high-level features from one-hot encoded sequences automatically before a set of dense layers and a softmax layer for classification. However, the following two issues hamper the performance of deep learning models for the recognition of short viral sequences as follows: (1) the single deep learning architecture suffers from failing to extract enough features to represent sequences; (2) the one-hot encoding strategy omits the relationship between two parts of a sequence because of its orthogonal property (Mikolov et al., 2013).

To solve the above-mentioned issues, a novel hybrid deep learning-based virus identifier, namely, DETIRE, is proposed in this study to identify viral fragments directly from metagenomes. DETIRE is a two-stage architecture, containing a graph convolutional network (GCN)-based sequence embedder and a two-path deep learning model. First, every sequence is cut into several 3-mer fragments, and, then successively input to the GCN-based sequence embedder to train the representations of all 3-mer fragments. After that, these embedded fragments are then fed into the CNN-path and BiLSTM-path to learn their features, respectively. Finally, by two dense layers and a softmax layer, pair scores are generated, and the higher score determines which type of the input sequence is.

The main contributions of this study are 2-fold: (1) the GCN-based embedder is utilized to enrich the representations of short sequences; (2) BiLSTM and CNN are combined to learn not only the spatial characteristics but also sequential characteristics simultaneously to generate abundant features of short sequences. To the best of our knowledge, DETIRE is the first hybrid model that combines CNN and BiLSTM networks to identify viruses from metagenomes.

The last of this study is organized as follows: Section 2 introduces the datasets used to train and test the DETIRE, and details the architecture of DETIRE and its training strategy. Section 3 shows the performance of the DETIRE on several datasets and discusses the selection of some key parameters which can affect the overall performance. Section 4 gives a brief conclusion of this study.

## 2. Materials and methods

### 2.1. Virus and host RefSeq genome datasets for training and testing

Virus RefSeq genome (up to 11 October 2022) was downloaded from NCBI Virus (https://www.ncbi.nlm.nih.gov/labs/virus/vssi/#/virus?SeqType_s=Genome&SourceDB_s=RefSeq). It has been proven that the less the difference between the lengths of sequences from the training and testing datasets, the better classification result will be achieved (Amgarten et al., 2018). Thus, all 34,492 viral sequences were split into a set of non-overlapped fragments with a length of 500 bp, resulting in 2,043,539 fragments total. The whole set of 500 bp viral fragments combined with 2,040,000 sequences of 500 bp subsampled from 4,410 prokaryotic host RefSeq genomes, supplied in VirFinder (Amgarten et al., 2018), called the GCN training dataset, was jointly used to train the GCN-based embedding model in order to generate a meaningful embedding matrix for sequences embedding. To show the ability of DETIRE for identifying new viruses, 400,000 viral fragments with a length of 500 bp from 22,066 viral sequences (before 16 August 2021) were subsampled randomly to be established as a training dataset to train a hybrid deep learning model. In total, 100,000 fragments from 6,188 viral sequences (between 16 August 2021 and 3 September 2022) were chosen randomly as a validation set. Moreover, 100,000 fragments from 6,238 viral sequences (between 3 September 2022 and 11 October 2022) were chosen randomly as a testing set. Since there is no overlap between the three datasets (training, validation, and testing datasets), a fully trained DETIRE may have the ability to identify new viral sequences (Jie et al., 2020; Shang and Sun, 2021). The NCBI accession numbers of the viral RefSeqs can be found on GitHub (https://github.com/crazyinter/DETIRE/blob/main/supplementary%20files/Virus_RefSeq_accession_numbers.csv).

### 2.2. Composition of DETIRE

**Figure 1:**
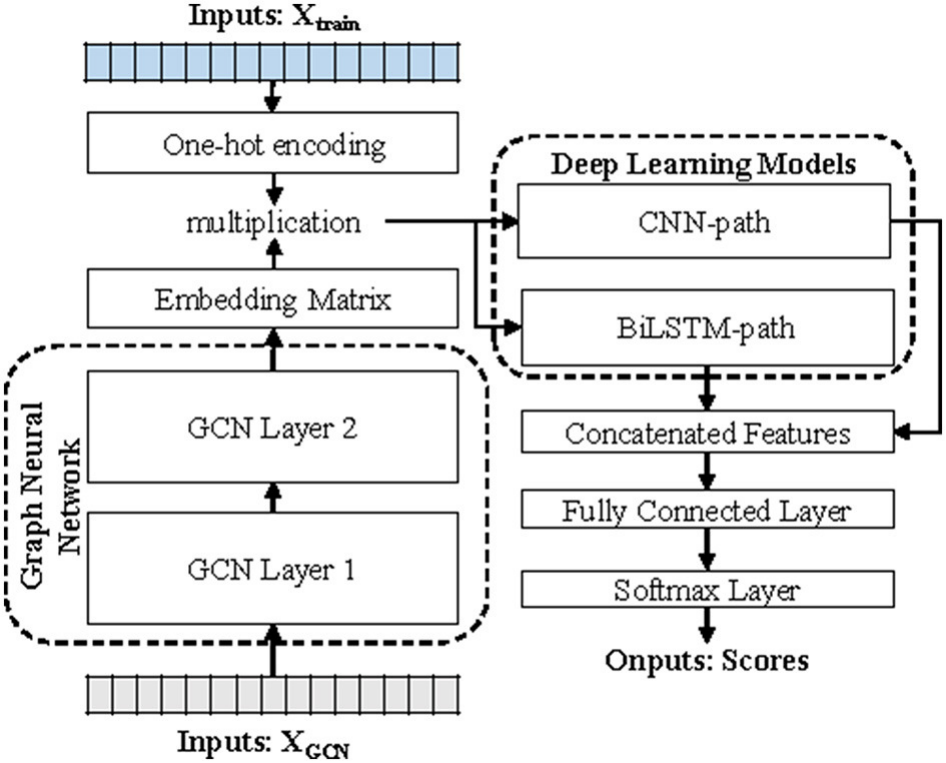
The workflow of DETIRE. DETIRE contains a GCN-based sequence embedding model and a deep learning-based method to learn features of viral sequences automatically and identify them directly from metagenomes. First, the graph neural network learns the high-level representations of 3-mer fragments in each sequence through supervised back propagation. Then, DETIRE extracts the features of spatial characteristics and sequential characteristics by designing the CNN model and LSTM model, respectively. Finally, the learned features are combined together to make the final decision by several dense layers and a softmax layer.

DETIRE utilizes a two-stage strategy for virus prediction, including GCN-based sequence embedding and deep learning-based sequence classification (Figure 1). Before embedding, every 3-mer fragment is generated by a three-base sliding window moving from the head to the tail of the sequence with a stride of one. For example, the original nucleotide sequence “ATTGCCTGACAT” will be cut into “ATT, TTG, TGC, GCC, CCT, CTG, TGA, GAC, ACA, and CAT.”

**Figure 2:**
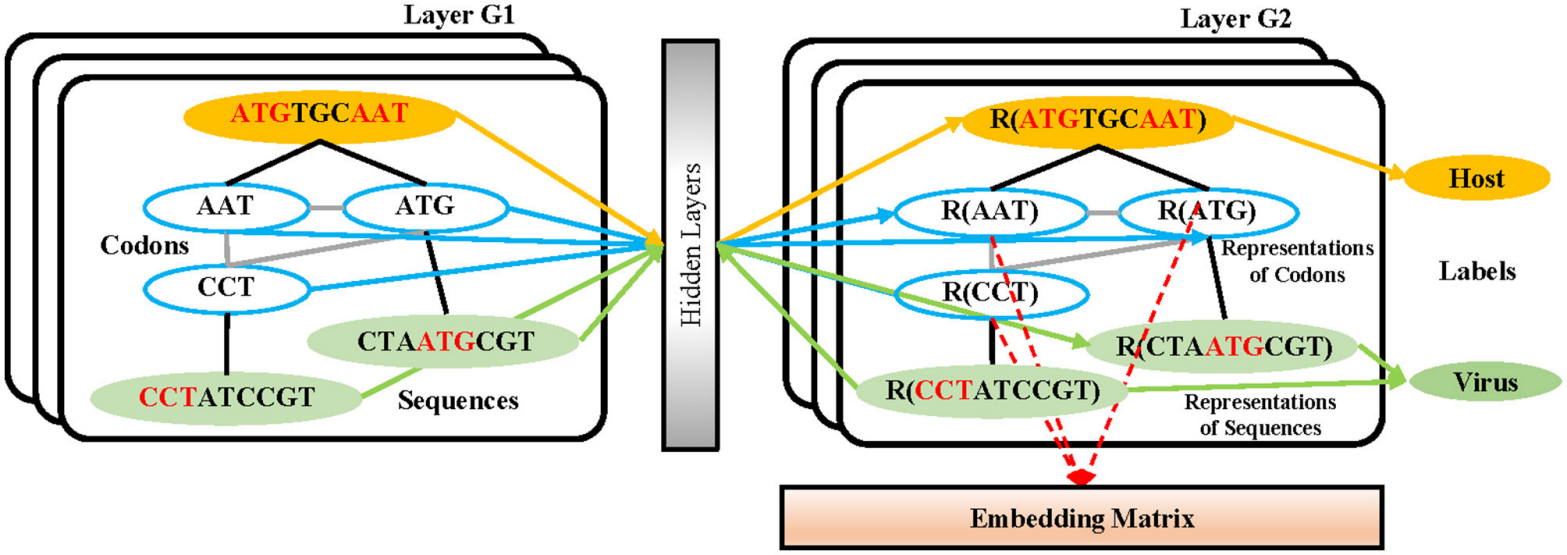
The structure of the GCN-based embedding model. Every nucleotide sequence in the GCN-training dataset and all unique 3-mer fragments from it are constructed to their single nodes. There are no edges between each nucleotide sequence. Edges are built between 3-mer fragments and their original sequences. All 64 3-mer fragments have an edge between each of them. After the training strategy, the vectors from the codon nodes in the second GCN layer are combined into an embedding matrix.

In the process of sequence embedding, TextGCN (Yao et al., 2019) is utilized to learn the meaningful high-level representations of all 3-mer fragments from every nucleotide sequence. First, a heterogeneous graph containing 3-mer fragment nodes is built in order to model global co-occurrence between these 3-mer fragments explicitly. Then, the built graph is fed into a simple two-layer GCN (Kipf and Welling, 2016). The first layer constructs the nodes and edges. Every nucleotide sequence in the GCN training dataset and all unique 3-mer fragments from it are constructed to their single nodes. There are no edges between each nucleotide sequence. Edges are built between 3-mer fragments and their original sequences. All 64 3-mer fragments have an edge between each of them (Figure 2). The weight of the edge between a sequence node and a 3-mer fragment node is determined by the term frequency-inverse document frequency (TF-IDF; Liang and Chengsheng, 2008) of the fragment in the sequence, where term frequency is the frequency of the 3-mer fragment appears in the sequence and inverse document frequency is the logarithmically scaled inverse fraction of the number of sequences that contain the 3-mer fragment. Point-wise mutual information (PMI; Church and Hanks, 1989), a popular measure for word associations, is employed to calculate weights between two fragment nodes. The second layer learns the fragment and sequence embeddings in each node. Finally, these nodes are fed into a softmax classifier, after which the cross-entropy error over all labeled sequences is defined as the cost function (Li et al., 2018). After 1,000 epochs of backpropagation by the Adam optimization algorithm (Kingma and Ba, 2015) with a learning rate of 0.015 and a dropout rate of 0.5, the 30-dimension representations of all 3-mer fragments in the second layer of the GCN are embedded into the sequences in the training and testing datasets.

In the process of sequence classification, two parallel deep learning models (a hybrid deep learning model), CNN and BiLSTM, respectively, used to learn spatial and sequential features of sequences. In the CNN path, each embedded sequence is considered as an image to extract a spatial feature through three sets of layers. Each set of the layer contains a convolutional layer (16, 32, and 64 filters with sizes of 7*7, 5*5, and 3*3, respectively), a ReLU activation function, a max pooling layer (with a pooling size of 4*4 and a stride of 4), and a batch normalization (BN) layer, respectively. In the BiLSTM path, the embedded 3-mer fragments in a sequence are input into the BiLSTM cells (498 tokens total) one by one, generating a sequential feature. Then, the first dense layer with 100 hidden neurons receives the weighted merged two sets of features from the CNN path and BiLSTM path. The second hidden layer after that contains 30 hidden neurons. Finally, a softmax layer generates two scores that reflect the likelihood of the input sequence as a virus or not. The weights of merging are two sets of trainable parameters which can be finetuned during the training progress. All of the parameters, here, are updated by Adam (Kingma and Ba, 2015) optimizer, with a mini-batch of 200 for 50 epochs to reduce the cross-entropy loss with a learning rate of 0.002.

### 2.3. Evaluation criteria

Universally, a confusion matrix is calculated to evaluate the performance of a classifier according to four statistics as follows: True Positives (TP), False Positives (FP), True Negatives (TN), and False Negatives (FN; Liu et al., 2022). TP are examples correctly labeled as positives; FP refer to negative examples incorrectly labeled as positive; TN correspond to negatives correctly labeled as negative; and FN refer to positive examples incorrectly labeled as negative. Several high-level criteria are further calculated based on the confusion matrix, such as recall, accuracy, precision, and F1 score:


(1) $$\begin{array}{l} Recall=\frac{TP}{TP+FN} \end{array}$$

(2) $$\begin{array}{l} Accuracy=\frac{TP+TN}{TP+FP+TN+FN} \end{array}$$

(3) $$\begin{array}{l} Precision=\frac{TP}{TP+FP} \end{array}$$

(4) $$\begin{array}{l} F1\;Score=\frac{2\times Precision\times Recall}{Precision+Recall} \end{array}$$

## 3. Results

### 3.1. A marine metagenome dataset from CAMI

To test the performance of DETIRE on identifying viral sequences, a marine metagenome was downloaded from the 2nd CAMI Challenge Marine Dataset (https://data.cami-challenge.org/participate), short and long-read shotgun metagenome data from samples at different seafloor locations of a marine environment. All sequences from the fasta document named CAMI2_short_read_pooled_gold_standard_assembly were selected. These sequences were, then, mapped to the Virus RefSeq genome using BLAST (Basic Local Alignment Search Tool) Altschul et al. (1990), with default parameters (word size is 11, threshold is 0, and score matrix is BLOSUM62). As a result, 8,431 sequences shorter than 500 bp, 7,087 sequences in length of 500–1,000 bp, 7,915 sequences in length of 1,000–3,000 bp, and 12,518 sequences longer than 3,000 bp were recognized as viral sequences. The same amount of non-viral short sequences was also randomly subsampled from the rest of sequences for each length, respectively. Together, these 71,902 mixed sequences were built into a metagenome dataset to test DETIRE.

### 3.2. A real human gut metagenome dataset

A real human gut metagenome dataset was downloaded from the NCBI short-read archive (accession ID: SRA052203; Sharon et al., 2013). The same BLAST progress with default parameters was conducted as it was in the 2nd CAMI Challenge Marine Dataset, resulting in 385 viral sequences shorter than 500 bp, 409 sequences in length of 500–1,000 bp, 728 sequences in length of 1,000–3,000 bp, and 915 sequences longer than 3,000 bp. Similarly, the same amount of non-viral short sequences for each length was subsampled randomly from the rest of non-viral sequences. Totally, 4,874 mixed sequences were used to test DETIRE.

### 3.3. The influence of k-mer fragments and their embedding sizes on the viral identification results

**Figure 3:**
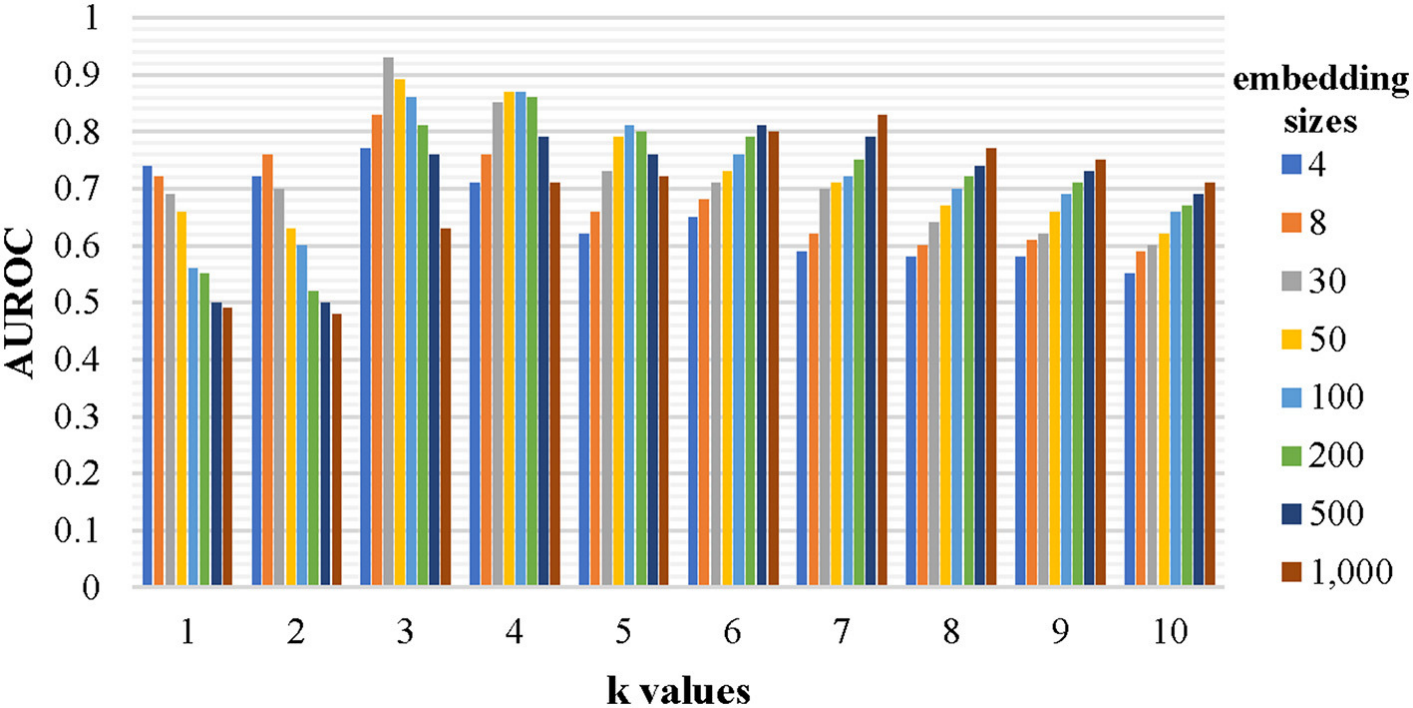
The performance of the models with various *k*-values and embedding sizes on the testing dataset. Sequences with specific *k*-mer fragments and embedding sizes were used to train several models, which were, then, evaluated by the testing dataset. The AUROC values of these models were calculated. The *k* is chosen from [1, 2, 3, 4, 5, 6, 7, 8, 9, and 10] and the embedding size is chosen from [4, 8, 30, 50, 100, 200, 500, and 1,000].

Every nucleotide sequence was converted into a set of *k*-mer fragments before being expressed as high-level vectors by the graph neural network to enrich the representation of the sequence. The chosen *k*-value and the embedding size have a significant impact on the identification performance. To find a relatively optimal set of the two parameters, a lot of models have been trained by the established training dataset and evaluated by the testing dataset. Each model corresponds to a specific set of parameters, where *k* is chosen from [1, 2, 3, 4, 5, 6, 7, 8, 9, and 10] and embedding size is chosen from [4, 8, 30, 50, 100, 200, 500, 1,000], respectively. The evaluation results are shown in Figure 3. When the embedding size is a little lower than 4 *k* (all combinations of the *k*-mer fragments), the AUROC value is close to the maximum. Thus, 3-mer fragments with an embedding size of 30 were chosen for our final model. The final representations of all 3-mer fragments can be found at https://github.com/crazyinter/DETIRE/blob/main/supplementary%20files/embedding_matrix.csv.

### 3.4. Performance on the testing dataset

To prove the outstanding performance of DETIRE on identifying short viral sequences (<500 bp), a testing experiment on the testing dataset was performed to make a comparison between DETIRE and three latest viral identification methods, DeepVirFinder, PPR-Meta, and CHEER. The accuracies, recalls, precisions, and F1 scores of the four methods are calculated in Table 1. DETIRE outperforms the three methods at all of the four criteria.

**Table 1:** Comparison of accuracies, recalls, precisions, and F1 scores of DeepVirFinder, PPR-Meta, CHEER, and DETIRE on the testing dataset.

**Criteria**	**Deep-VirFinder**	**PPR-Meta**	**CHEER**	**DETIRE**
Accuracy	0.8126	0.8403	0.8744	**0.8772**
Recall	0.8159	0.8396	0.8782	**0.8812**
Precision	0.8105	0.8408	0.8715	**0.8741**
F1 score	0.8132	0.8402	0.8748	**0.8776**

Bold values represent the best performance.

### 3.5. Performance on the CAMI Marine metagenome

To deal with different lengths of sequences from the metagenome and avoid vanishing gradient problem in the process of training BiLSTM, a sequence longer than 500 bp will be divided into several non-overlapped sub-sequences of 500 bp before input into the BiLSTM path. If the length of the last part in the sequence is shorter than 500 bp, the last bases of the sequence will be zero-padded and regarded as a single subsequence. Then, all of the sub-sequences are input to the hybrid deep learning model one after another, to get their own scores, the average of which will be the final score and is contributed to identifying whether the query long sequence is viral or not.

The accuracies, recalls, precisions, and F1 scores of DETIRE, CHEER, PPR-Meta, and DeepVirFinder on classifying viral and non-viral sequences from the CAMI Marine metagenome are calculated and made a comparison in Table 2. DETIRE exceeds DeepVirFinder and PPR-Meta at all of the four criteria for identifying all lengths of viral sequences. DETIRE is better than CHEER when the length is shorter than 3,000 bp. For identifying long sequences (>3,000 bp), the accuracy, recall, and F1 score of DETIRE are slightly lower than CHEER because DETIRE identified a little less viral sequences. For all lengths, DETIRE achieves the best performance than the other three methods.

**Table 2:** Comparison of accuracies, recalls, precisions, and F1 scores of DeepVirFinder, PPR-Meta, CHEER, and DETIRE on the CAMI Marine metagenome.

**Length**	**Criteria**	**Deep-VirFinder**	**PPR-Meta**	**CHEER**	**DETIRE**
<500 bp	Accuracy	0.7840	0.7982	0.7982	**0.8061**
	Recall	0.7860	0.8009	0.8041	**0.8109**
	Precision	0.7828	0.7967	0.7948	**0.8032**
	F1 score	0.7844	0.7988	0.7994	**0.8071**
500–1,000 bp	Accuracy	0.8130	0.8435	0.9007	**0.9030**
	Recall	0.8074	0.8369	0.9001	**0.9040**
	Precision	0.8165	0.8481	0.9012	**0.9021**
	F1 score	0.8119	0.8425	0.9007	**0.9031**
1,000–3,000 bp	Accuracy	0.8269	0.8400	0.8956	**0.8964**
	Recall	0.8221	0.8358	0.8964	**0.8973**
	Precision	0.8301	0.8429	0.8947	**0.8957**
	F1 score	0.8261	0.8393	0.8956	**0.8965**
>3,000 bp	Accuracy	0.8409	0.8519	**0.8708**	0.8699
	Recall	0.8434	0.8485	**0.8724**	0.8691
	Precision	0.8393	0.8544	0.8696	**0.8706**
	F1 score	0.8413	0.8514	**0.8710**	0.8698
Overall	Accuracy	0.8194	0.8351	0.8652	**0.8673**
	Recall	0.8190	0.8322	0.8672	**0.8685**
	Precision	0.8196	0.8370	0.8637	**0.8664**
	F1 score	0.8193	0.8346	0.8654	**0.8675**

Bold values represent the best performance.

### 3.6. Performance on the real human gut metagenome

The accuracies, recalls, precisions, and F1 scores of DETIRE, CHEER, PPR-Meta, and DeepVirFinder are calculated according to the number of correctly and incorrectly identified viral and host sequences from the real human gut metagenome dataset (Table 3). DETIRE also achieves the best overall performance than the other three methods of identifying viral sequences (all lengths). In spite of a 0.0087 and 0.0019 lower precision than CHEER for 500–1,000 bp and 1,000–3000 bp, DETIRE gets higher accuracy, recall, and F1 score for these lengths. For sequences longer than 3,000 bp, CHEER is the outperforming method. However, a comparable performance of DETIRE is achieved as CHEER for long sequences.

**Table 3:** Comparison of accuracies, recalls, precisions, and F1 scores of DeepVirFinder, PPR-Meta, CHEER, and DETIRE on the real human gut metagenome.

**Length**	**Criteria**	**Deep-VirFinder**	**PPR-Meta**	**CHEER**	**DETIRE**
<500 bp	Accuracy	0.8299	0.8052	0.8831	**0.8905**
	Recall	0.8234	0.8026	0.8857	**0.8962**
	Precision	0.8342	0.8068	0.8811	**0.8828**
	F1 score	0.8288	0.8047	0.8834	**0.8894**
500–1,000 bp	Accuracy	0.8154	0.8154	0.8863	**0.8924**
	Recall	0.8093	0.8044	0.8875	**0.8998**
	Precision	0.8193	0.8225	**0.8954**	0.8867
	F1 score	0.8143	0.8133	0.8864	**0.8932**
1,000–3,000 bp	Accuracy	0.8592	0.8482	0.8860	**0.8874**
	Recall	0.8722	0.8448	0.8777	**0.8832**
	Precision	0.8501	0.8506	**0.8925**	0.8906
	F1 score	0.8610	0.8477	0.8850	**0.8869**
>3,000 bp	Accuracy	0.8317	0.8404	**0.8650**	0.8596
	Recall	0.8393	0.8481	**0.8732**	0.8634
	Precision	0.8267	0.8353	**0.8591**	0.8568
	F1 score	0.8330	0.8416	**0.8661**	0.8601
Overall	Accuracy	0.8369	0.8330	0.8777	**0.8818**
	Recall	0.8416	0.8326	0.8789	**0.8842**
	Precision	0.8337	0.8333	0.8768	**0.8800**
	F1 score	0.8377	0.8329	0.8779	**0.8821**

Bold values represent the best performance.

### 3.7. Comparison of the testing time consuming

The testing time of the four methods on the testing dataset, the CAMI Marine metagenome and the real human gut metagenome are made a comparison, as shown in Table 4. The equipment used for the analysis is two Intel Xeon Gold 6226R (CPU) with a memory of 256 Gb. For all the three datasets, DETIRE has the minimum time consumption for the testing strategies.

**Table 4:** Comparison of the testing time consuming on the three datasets.

**Datasets**	**Deep-VirFinder**	**PPR-Meta**	**CHEER**	**DETIRE**
Testing dataset(s)	762	812	891	**716**
CAMI Marine(s)	362	321	332	**304**
Real human gut(s)	130	114	121	**112**

Bold values represent the best performance.

### 3.8. Availability of DETIRE on identifying phages

DETIRE is trained to identify viral sequences directly from metagenomes, including prokaryotic virus and eukaryotic virus. In some specific virus analysis tasks, finding bacteriophage-originated sequences is a meaningful strategy. To test the ability of DETIRE to detect phages, a phage dataset was established according to DeePhage Wu et al. (2021). All 225 phage genomes were collected, and then, 10 fragments of 500 bp were randomly subsampled from each phage genome, resulting in 2,250 sequences.These sequences were embedded by the trained embedding matrix and input to DETIRE. DeepVirFinder, PPR-Meta, and CHEER were also tested by these sequences. The accuracies of identifying these phages are shown in Table 5. DETIRE identified 90.27% phages and 4.36 and 4.58% more than CHEER and DeepVirFinder, respectively. The accuracy of DETIRE is 2.49% lower than PPR-Meta. This may be caused by PPR-Meta's training dataset, containing only phages because it is a 3-class classifier that allows simultaneous identification of both phage and plasmid fragments. Eventhough, DETIRE achieves a better performance than CHEER and DeepVirFinder, with relatively comparable results as PPR-Meta. This means DETIRE has the potential in identifying phages.

**Table 5:** Comparison of accuracies on identifying phages.

**Methods**	**Deep-VirFinder**	**PPR-Meta**	**CHEER**	**DETIRE**
Accuracy (%)	85.69	**92.76**	85.91	90.27

Bold values represent the best performance.

### 3.9. The ability of DETIRE on making a whole viral genome by identifying viral sequences after assembling

DETIRE is tested additionally by metagenomic data with known viral infection, to see if the identified viral sequences can be assembled to make whole genome of the virus. A wastewater metagenome (Schoch et al., 2020; NCBI:txid527639) is selected from the IMG/VR database David et al. (2021), containing 359 viral nucleotide sequences. All of the sequences are separated into 100 bp sequences, resulting in 3,459 reads. All of the four methods were separately used to identify these reads as virus or not. The number of identified viral sequences and accuracy is shown in Table 6. Then, these identified viral sequences are assembled by SOAPdenovo2 Ruibang et al. (2012), with default parameters. The number of assembled contigs is shown in Table 6. After that, these contigs are input to CheckV Nayfach et al. (2021), to assess the quality and completeness of the viral genome. The number of hits for each method is shown in Table 6. All the hits are of low quality by the AAI-based method. The completeness of contigs from DETIRE can be seen in https://github.com/crazyinter/DETIRE/blob/main/supplementary%20files/DETIRE_completeness.csv. DETIRE identifies most viral reads from the wastewater metagenome. A total of 289 assembled viral contigs are detected by CheckV, with low-quality, 24, 88, and 77 more than DeepVirFinder, PPR-Meta, and CHEER, respectively. The results show that DETIRE performs better than the other three methods of making whole genome of the virus by assembling identified viral sequences.

**Table 6:** The results of four methods on making whole viral genome by identified viral sequences after assembling.

**Criteria**	**Deep-VirFinder**	**PPR-Meta**	**CHEER**	**DETIRE**
Number of identified reads	1,352	1,402	2,126	**2,203**
Accuracy	0.3909	0.4053	0.6146	**0.6369**
Number of assembled contigs	561	568	873	**890**
Number of hits	212	201	265	**289**

Bold values represent the best performance.

## 4. Discussions

Currently, identifying short viral sequences directly from metagenomes is still of low accuracy because of too little information to extract enough features from short sequences. There are three reasons why DETIRE works. First, the GCN-based embedding model creates a new representation for a sequence. Each codon is transformed into a high-dimensional embedded vector by a trained embedding matrix. The new representation of the sequence enriches the features significantly. Second, the hybrid model contains BiLSTM and CNN simultaneously. It could learn not only the spatial characteristics but also sequential characteristics to generate abundant features of short sequences, which is intuitively consistent with the expression of the DNA sequence in nature. Thirdly, the combination of the two features extracted from the CNN-path and BiLSTM-path is dynamic. The longer the input sequence, the more contribution of the CNN-path. Similarly, the shorter the input sequence, the more contribution of the BiLSTM-path. Furthermore, the attention mechanism in both paths decides which part of the information from the sequence should be remembered or forgotten, which is really important to determining which part of the sequence contributes more to distinguishing.

**Figure 4:**
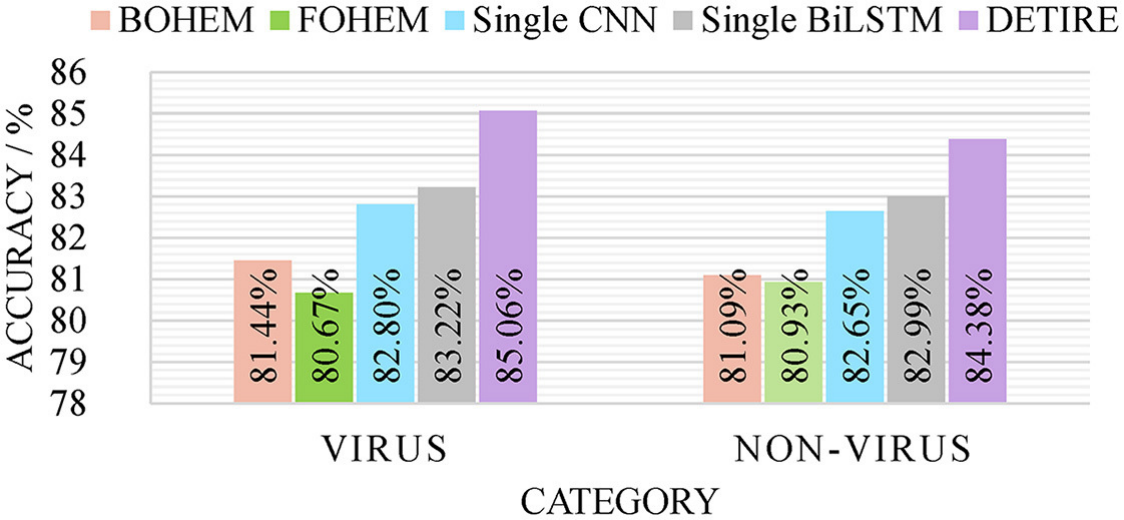
The identification result of BOHEM, FOHEM, single CNN, single BiLSTM, and DETIRE on the testing dataset. The five models are tested by the testing dataset to prove the effectiveness of the GCN-based sequence embedding method and the hybrid deep learning based-architecture.

To verify the effectiveness of the GCN-based embedding model and hybrid classification model in DETIRE, the sequences from the same training and testing datasets (500 bp) were one-hot encoded in two separate ways as follows: each base in a sequence was one-hot encoded to [1,0,0,0], [0,1,0,0], [0,0,1,0], or [0,0,0,1]; each 3-mer fragment in a sequence was one-hot encoded to a 64-dimension vector. Then, the two one-hot encoded training datasets were used to train two hybrid models, namely, BOHEM (base one-hot encoded model) and FOHEM (3-mer fragments one-hot encoded model). The two models both have the same BiLSTM-path and CNN-path as DETIRE. Another single CNN model and another single BiLSTM model were trained and tested separately by the same training and testing datasets. The sequences in the datasets were all embedded by the trained embedding matrix in DETIRE. Each model has the same hyper-parameters as the corresponding deep learning model in DETIRE. All parameters were finetuned during the training strategy. The four models are tested by the testing datasets and made a comparison with DETIRE, which is shown in Figure 4. The accuracy of DETIRE in identifying viral sequences exceeds that of BOHEM and FOHEM by 3.62 and 4.39%, respectively, representing the effectiveness of the GCN-based sequence embedding method in DETIRE. DETIRE also has a superiority over single CNN-based and BiLSTM-based model. The gap is 2.26 and 1.74% on identifying viral sequences, respectively, showing the availability of the hybrid deep learning model in DETIRE.

The advantage of DETIRE becomes smaller with the increase in the length of input sequences. This may be caused by the length of sequences in the GCN-training dataset being 500 bp. The trained embedding matrix is more suitable for identifying short viral sequences. Furthermore, the labels of the sequences in the marine metagenome dataset and the real human gut metagenome dataset are made by the BLAST search in the whole virus RefSeq. This may lead to false positives or negatives for viruses because the training set for the hybrid deep learning model is subsampled from the virus RefSeq. However, the size of the training set is rather smaller than the whole virus RefSeq, and the influence of the duplicate data would be rather small.

DETIRE could accurately identify short viral sequences (<1,000 bp) from metagenomes, with comparable performance at longer length (>1,000 bp). It supplements viral analysis since a high proportion of the existing tools tend to deal with long sequences generated from assembling and binning, which misses many viral species. Moreover, identifying viral sequences is the very first step in the viral analysis, the effectiveness of which could have an impact on downstream work, such as virus taxonomy, virus–host interactions, and virus-derived diseases detection. We hope that DETIRE would play an important role in the realm of virus analysis.

## 5. Conclusion

In this study, a deep learning-based hybrid model, DETIRE, is proposed to identify viral sequences directly from the metagenome. Encoded by a graph-based embedding method, nucleotide sequences are fed into a CNN-path and a BiLSTM-path, respectively, for feature extraction before being classified by a softmax layer. In comparison to the three latest viral identification methods, DeepVirFinder, PPR-Meta, and CHEER, on the test dataset, the CAMI Marine dataset and a real human gut metagenome, DETIRE outperforms in identifying short sequences (<1,000 bp). DETIRE will do something to promote the development of the field on the natural viral community analysis because of the huge number of short sequences generated by the NGS technique. DETIRE is anticipated to play a positive role in the downstream viral analysis such as viral taxonomy and pathogens identification.

## Data availability statement

The original contributions presented in the study are included in the article/supplementary material, further inquiries can be directed to the corresponding author.

## Author contributions

YM proposed the method and wrote the manuscript. YM and TD conceived the experiments and analyzed the results. JB helped to retrain DETIRE and add some technical tricks to improve the performance of DETIRE. GD compared the four methods on the impact of false positive assembly. All authors reviewed the manuscript.
